# Multiple cerebral microbleeds and atypical β-amyloid deposits: A case report

**DOI:** 10.1097/MD.0000000000018296

**Published:** 2019-12-20

**Authors:** Jin San Lee, Kyung Mi Lee, Hyug-Gi Kim, Il Ki Hong

**Affiliations:** a Department of Neurology; b Department of Radiology; c Department of Nuclear Medicine, Kyung Hee University Hospital, Kyung Hee University College of Medicine, Seoul, South Korea.

**Keywords:** amyloid deposition, cerebral microbleeds, cognitive impairment

## Abstract

**Rationale:**

Cerebral microbleeds are increasingly recognized in various neurological disorders such as cerebral amyloid angiopathy (CAA), Alzheimer disease, and stroke. The presence and number of cerebral microbleeds are known to be independent predictors of cognitive impairment.

**Patient concerns:**

A 73-year-old woman visited our memory disorder clinic complaining of progressive memory impairment, which started 2 years ago.

**Diagnoses:**

The patient had innumerable cortical/subcortical cerebral microbleeds in the entire brain. We diagnosed the patient with amnestic mild cognitive impairment due to CAA. Interestingly, only focal β-amyloid deposits at the bilateral parietal cortices were seen on amyloid positron emission tomography (PET) scan.

**Interventions:**

We have observed changes in her cognitive function without any medication.

**Outcomes:**

The cognitive function of the patient was unchanged during the follow-up period.

**Lessons:**

Our case was interesting in a few aspects, including the number of cerebral microbleeds and the atypical β-amyloid deposition pattern on amyloid PET scan. Further studies on more cases are needed to evaluate β-amyloid burden and distribution patterns in CAA.

## 1 Introduction

Cerebral microbleeds are defined as small (≤2 mm), round dark-signal lesions on T2∗ gradient-echo or susceptibility-weighted imaging (SWI) magnetic resonance imaging (MRI) and frequently found in the general elderly population. Growing evidence suggests that cerebral microbleeds are associated with 2 types of small vessel disease: hypertensive arteriopathy and cerebral amyloid angiopathy (CAA).^[1]^ In CAA, cerebral microbleeds are preferentially located in the lobar area. Vascular amyloid in CAA leads to various types of brain damage, including focal and more widespread parenchymal brain injury. Sporadic CAA is known as a potential contributor to age- and Alzheimer's disease (AD)-related cognitive impairment.^[2,3]^ Also, the presence and number of cerebral microbleeds are known to be independent predictors of cognitive impairment.^[4]^ We report an interesting case of a patient with innumerable cerebral microbleeds and atypical β-amyloid deposits.

### 1.1 Consent statement

Written informed consent was obtained from the patient for the publication of this case report.

## 2 Case presentation

A 73-year-old left-handed woman with 6 years of education was brought to our memory disorder clinic by her daughter who reported that the patient had developed progressive memory impairment over the past 2 years. The daughter reported that the patient did not have any history of depression or psychiatric problems. There was no preceding history of head trauma, encephalitis, antipsychotic or antiemetic medication use, or exposure to toxic substances. The patient had been diagnosed with hypertension 5 years previously, and was taking antihypertensive medication at the time of visiting our clinic.

On neurological examination, her Mini-Mental State Examination score (MMSE) was 24/30 (1 point lost on orientation to time, 3 points lost on attention and calculation task, and 2 points lost on recall of the 3 items). The cranial nerves were intact and motor examination revealed normal muscle strength and tone; deep tendon reflexes were also normal. Sensory examination results were also normal for all modalities. The Babinski sign was not elicited. Detailed neuropsychological tests revealed impairment of verbal memory and frontal/executive functions, but no impairment of attention, language, visual memory, and visuospatial functions. Moreover, there was no evidence of impaired activities of daily living. In addition, no neuropsychiatric symptoms were noted on the neuropsychiatric inventory questionnaire. Her score on the Geriatric Depression Scale was 2/30.

**Figure 1:**
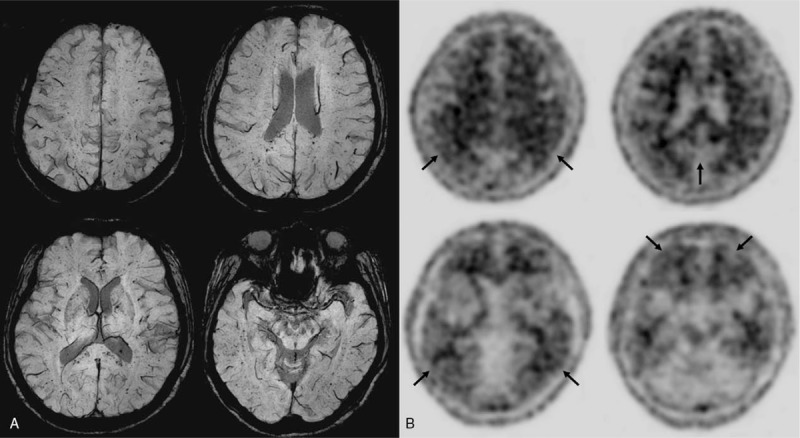
(A) Brain MRI (SWI sequence). There are innumerable cerebral microbleeds at the bilateral frontal, parietal, temporal, and occipital lobes. (B) The ^18^F-florbetaben PET images. Visual assessment of the four main target regions based on RCTU and BAPL scoring judged as positive for β-amyloid plaques (BAPL 3, parietal = RCTU 3; posterior cingulate/precuneus = RCTU 1; lateral temporal = RCTU 1; frontal = RCTU 1). BAPL = brain β-amyloid plaque load, MRI = magnetic resonance imaging, PET = positron emission tomography, RCTU = regional cortical tracer uptake, SWI = susceptibility weighted imaging.

Serological and hematological tests, including complete blood count, blood chemistry, vitamin B12/folate, and thyroid function tests yielded normal results. Rapid plasma reagin and *Treponema pallidum* hemagglutination assays also yielded normal results. However, brain MRI demonstrated innumerable cerebral microbleeds at the bilateral frontal, parietal, temporal, and occipital lobes on SWI sequence (Fig. 1A). There were mild white matter hyperintensities but no lacunes or enlarged perivascular spaces on fluid-attenuated inversion recovery and T2-weighted imaging. Scheltens visual rating scale score for medial temporal lobe atrophy was 1/1 (Right/Left). There were no structural lesions including territorial cerebral infarction, brain tumors, hippocampal sclerosis, and vascular malformation. We diagnosed the patient with amnestic mild cognitive impairment (MCI) based on the proposed criteria for MCI.^[5]^ In addition, probable CAA was diagnosed in this patient according to the consensus criteria.^[6]^

Since her apolipoprotein E genotype revealed ε3/ε4 and probable CAA was suspected, we performed ^18^F-florbetaben positron emission tomography (PET) to evaluate β-amyloid deposition in the brain. Visual assessment of the ^18^F-florbetaben PET images revealed a positive scan for β-amyloid plaques based on regional cortical tracer uptake (RCTU) and brain β-amyloid plaque load (BAPL) scoring (Fig. 1B). However, in contrast to the diffuse cortical β-amyloid deposition pattern usually observed in AD, only focal β-amyloid deposits at the bilateral parietal cortices were seen in this patient (RCTU 3).

According to the recently proposed guideline on MCI,^[7]^ we counseled the patient and family that there are no pharmacologic or dietary agents currently shown to have symptomatic cognitive benefit in MCI. We recommended regular exercise (twice/week) and cognitive training to the patient. At 1-year follow-up, the cognitive function of the patient, as assessed by MMSE, remained unchanged (24/30).

## 3 Discussion

Based on these neuroimaging findings and the cognitive profile of the patient, we diagnosed her with amnestic MCI due to probable CAA. Amnestic MCI is regarded as an intermediate state between normal aging and dementia, especially AD.^[8]^ The clinical outcomes of MCI patients are heterogeneous, as some patients quickly progress to AD dementia while others remain stable or even revert to normal cognition.^[9]^ To date, there are no specific treatments for cognitive symptoms in MCI. However, in patients with MCI, regular exercise and cognitive interventions may be beneficial in improving cognitive function according to the recently proposed guideline on MCI.^[7]^

Our case was interesting in a few aspects, including the number of cerebral microbleeds and the atypical β-amyloid deposition pattern. First, the patient had innumerable cortical/subcortical cerebral microbleeds in the brain. In a community-based sample of older people without dementia at baseline, there was suggestive evidence of a higher rate of incident dementia in people with multiple cerebral microbleeds.^[10]^ However, although our patient had innumerable cerebral microbleeds, her cognitive status was amnestic MCI, not dementia. Her age and MRI findings fulfilled the criteria for probable CAA. However, we assume that her cognitive function was relatively maintained since total burden of cerebral small vessel disease,^[2]^ such as white matter hyperintensities, lacunes, cortical superficial siderosis, or enlarged perivascular spaces, was not too high. Careful follow-up is necessary to observe changes in her cognitive function.

Another noteworthy finding, in this case, was the atypical β-amyloid deposition pattern on an amyloid PET scan. The regional cerebral microbleeds distribution seen in CAA is mainly located in the posterior lobar brain regions (especially the occipital lobes and the temporal-parietal regions).^[11]^ The pathological hallmark of CAA is progressive β-amyloid deposition affecting the cortical and leptomeningeal vessels. The vessels affected by CAA can show vasculopathic changes, including fibrinoid necrosis, microaneurysm formation, wall thickening, and deposition of perivascular blood-breakdown products.^[12]^ During the course of the disease, both cerebrovascular and parenchymal β-amyloid deposition may coexist. A recent meta-analysis has reported that the occipital lobe is most frequently and severely affected by CAA, and higher cerebrovascular and parenchymal β-amyloid deposits were found in this region.^[13]^ Therefore, the case presented here is a deviation from what has been previously reported because only focal β-amyloid deposits at the bilateral parietal cortices were noted on amyloid PET scan, although the patient had innumerable cortical/subcortical cerebral microbleeds in the entire brain. Further studies on more cases are needed to evaluate β-amyloid burden and distribution patterns in CAA.

## Author contributions

**Conceptualization:** Jin San Lee, Kyung Mi Lee.

**Investigation:** Jin San Lee, Kyung Mi Lee, Hyug-Gi Kim, Il Ki Hong.

**Resources:** Jin San Lee, Il Ki Hong.

**Writing – original draft:** Jin San Lee, Kyung Mi Lee, Hyug-Gi Kim, Il Ki Hong.

**Writing – review & editing:** Jin San Lee, Kyung Mi Lee.

Kyung Mi Lee orcid: 0000-0003-3424-0208.
